# Sigma-1 Receptor Agonist Fluvoxamine Ameliorates Fibrotic Response of Trabecular Meshwork Cells

**DOI:** 10.3390/ijms241411646

**Published:** 2023-07-19

**Authors:** Judit Hodrea, Minh Ngoc Tran, Balazs Besztercei, Timea Medveczki, Attila J. Szabo, Laszlo Orfi, Illes Kovacs, Andrea Fekete

**Affiliations:** 1 MTA-SE Lendület “Momentum” Diabetes Research Group, Semmelweis University, 1083 Budapest, Hungaryngocminhdt@gmail.com (M.N.T.);; 2 Pediatric Center, MTA Center of Excellence, Faculty of Medicine, Semmelweis University, 1083 Budapest, Hungary; 3 Department of Biochemistry, University of Medicine and Pharmacy at Ho Chi Minh City, Ho Chi Minh City 72712, Vietnam; 4 Institute of Clinical Experimental Research, Semmelweis University, 1094 Budapest, Hungary; 5 Department of Pharmaceutical Chemistry, Semmelweis University, 1092 Budapest, Hungary; 6 Department of Ophthalmology, Semmelweis University, 1085 Budapest, Hungary; 7 Department of Clinical Ophthalmology, Faculty of Health Sciences, Semmelweis University, 1085 Budapest, Hungary

**Keywords:** sigma-1 receptor, fluvoxamine, fibrosis, trabecular meshwork, PDGF

## Abstract

Primary open-angle glaucoma remains a global issue, lacking a definitive treatment. Increased intraocular pressure (IOP) is considered the primary risk factor of the disease and it can be caused by fibrotic-like changes in the trabecular meshwork (TM) such as increased tissue stiffness and outflow resistance. Previously, we demonstrated that the sigma-1 receptor (S1R) agonist fluvoxamine (FLU) has anti-fibrotic properties in the kidney and lung. In this study, the localization of the S1R in TM cells was determined, and the anti-fibrotic efficacy of FLU was examined in both mouse and human TM cells. Treatment with FLU reduced the F-actin rearrangement, inhibited cell proliferation and migration induced by the platelet-derived growth factor and decreased the levels of fibrotic proteins. The protective role of the S1R in fibrosis was confirmed by a more pronounced increase in alpha smooth muscle actin and F-actin bundle and clump formation in primary mouse S1R knockout TM cells. Furthermore, FLU demonstrated its protective effects by increasing the production of nitric oxide and facilitating the degradation of the extracellular matrix through the elevation of cathepsin K. These findings suggest that the S1R could be a novel target for the development of anti-fibrotic drugs and offer a new therapeutic approach for glaucoma.

## 1. Introduction

Glaucoma is the second most common cause of blindness, affecting nearly 80 million people worldwide, and this is projected to increase to 111,8 million by 2040 [1,2,3,4]. Primary open-angle glaucoma (POAG) is the most common form of glaucoma that is characterized by increased intraocular pressure (IOP) and the accelerated loss of retinal ganglion cells. IOP is the main risk factor of the disease [5,6,7,8], and it is determined by the balance between aqueous humor production by the ciliary body and its outflow via two drainage routes: the trabecular meshwork (TM) outflow and the uveoscleral outflow pathway, with the latter accounting for a smaller proportion. While increased IOP can result from both, increased resistance to outflow and elevated tissue resistance due to the progressive fibrosis of the TM serve as the primary cause behind the development of glaucoma [9].

Although many pharmaceutical agents have been developed to reduce IOP, the treatment of glaucoma continues to be challenging due to the refractory nature of the disease to these available agents or the occurrence of significant adverse effects [10,11,12]. Consequently, there remains a constant medical need for novel therapies, particularly for new compounds that directly target the dysfunction of the TM tissue.

The molecular mechanisms controlling TM outflow are not well understood, but damage certainly occurs at the cellular level. The platelet-derived growth factor (PDGF) is a well-described factor that drives fibrosis in almost all organs [13], and its blockage might therefore be a potential therapeutic approach for pathological fibrotic diseases. Fibrotic TM tissue is associated with glaucoma [9,14]. Therefore, targeting TM fibrosis is an attractive strategy for therapeutic intervention in POAG.

The sigma-1 receptor (S1R) is a small transmembrane protein that is vastly expressed in the central nervous system, and its neuro-protective effect has been widely investigated, including the retina. Recently, we demonstrated that S1R activation with fluvoxamine exhibits anti-fibrotic properties in peripheral organs such as the kidney and lung [15]. These findings suggest that S1R activation can serve as a novel treatment approach for tissue scarring associated with excessive extracellular matrix (ECM) deposition.

The aim of this study is to determine the localization of the S1R in the TM and validate in vitro S1R activation as a potential anti-fibrotic target for future treatment of fibrosis-associated TM diseases, particularly POAG.

## 2. Results

### 2.1. S1R Resides in the Endoplasmic Reticulum (ER) Membrane and in the Cytoplasm of Human Trabecular Meshwork Cells (HTM5), and Its Selective and Specific Agonist Fluvoxamine (FLU) Enters the Cells In Vivo

**Figure 1:**
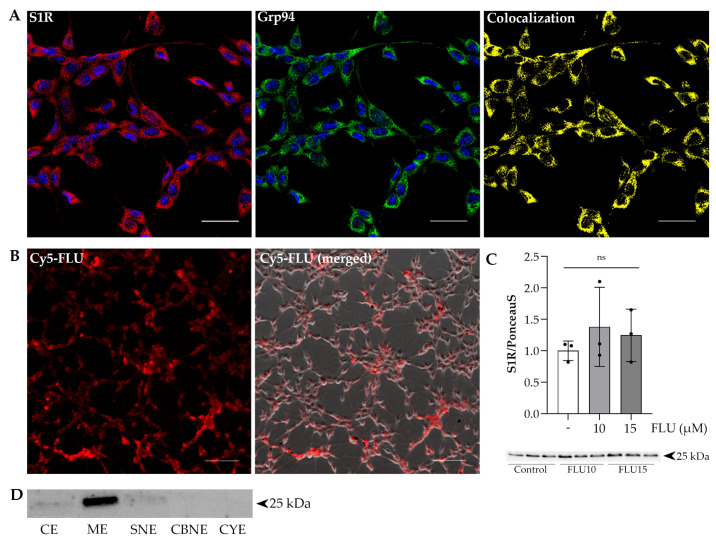
Cellular localization and Western blot of sigma-1 receptor (S1R) in human trabecular meshwork (HTM5) cells. (**A**) S1R localizes in the endoplasmic reticulum of HTM5 cells (S1R: red; Grp94: green; nuclei: blue; colocalization of S1R and Grp94: yellow). (**B**) Cy5-FLU (500 nM) penetrates live HTM5 cells (Cy5-FLU: red). (Nikon Eclipse Ti2 microscope; magnification: (**A**) 600×; (**B**) 200×; scale bar: (**A**) 20 µm; (**B**) 100 µm.) (**C**) Representative Western blot of S1R (25 kDa) protein of HTM5 cells. An amount of 10 µM or 15 µM of S1R agonist FLU does not affect S1R protein level of HTM5. (**D**) Subcellular localization of S1R in HTM5 cells (CE: Cytoplasmic extract; ME: Endoplasmic reticulum membrane extract; SNE: Soluble nuclear extract; CBNE: Chromatin-bound nuclear extract; CYE: Cytoskeletal extract) (data: mean ± SEM; n = 3/group; ns: non-significant; ANOVA followed by Holm–Šidak multiple comparison test).

Recent studies report on its function and distribution in retinal cells [16,17,18,19,20,21,22,23]. However, there is no data on its localization in trabecular meshwork cells. As first investigators, we visualized the S1R and analyzed its co-localization with the ER marker Grp94 (Figure 1A). A Pearson correlation coefficient of 0.784 and Spearman’s coefficient of 0.931 strongly support that the S1R resides mainly in the ER membrane and is also found in a lesser amount in the cytoplasm of HTM5 cells. Western blot analysis of proteins from various cellular compartments obtained after subcellular protein fractionation also supports these findings. The S1R is predominantly found in the ER membrane, with a small amount in the cytoplasmic (CE) fraction and a minimal presence in the soluble nuclear (SNE) protein fraction (Figure 1D).

The presence of FLU in living cells was confirmed by live cell imaging using an exclusively designed, custom-made, Cyanine5 far-red-fluorescent-dye-tagged FLU (Cy5-FLU). This staining was observed to be prominent, indicating that FLU can indeed cross the cell membrane and penetrate TM cells (Figure 1B). We also showed that under normal conditions, treatment with FLU did not increase the receptor’s protein level (Figure 1C).

For the cell characterization, the induction of myocilin in response to dexamethasone (Dex), as the most reliable marker for the trabecular meshwork [24,25,26], was investigated. In addition, other behaviors, such as the Dex-induced production of ECM elements, have also been observed in TM cells [25,27,28]. As expected, myocilin increased, furthermore, fibronectin, and alpha smooth muscle actin (α-SMA) levels were enhanced as well (Figure S3A–D). These data, which are in line with the literature, confirmed that the cells are truly trabecular meshwork cells.

### 2.2. FLU Ameliorates the PDGF-Induced F-Actin Enhancement in Human Trabecular Meshwork (HTM5) Cells

**Figure 2:**
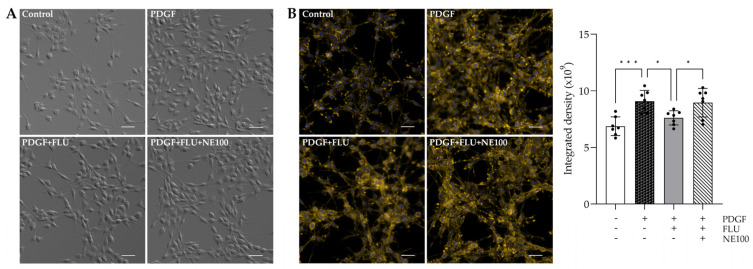
F-actin architecture in human trabecular meshwork (HTM5) cells after PDGF, fluvoxamine (FLU) and NE100 treatment. (**A**) FLU moderates the morphological changes in PDGF-induced HTM5 cells. (**B**) F-actin filaments and clump formation are ameliorated by FLU treatment. In both cases, NE100 suspends the effect of FLU. Quantification of fluorescence is presented as integrated density (F-actin: yellow; nuclei: blue; Nikon Eclipse Ti2 microscope; magnification: 200×; scale bar: 20 µm; data: mean ± SEM; n = 7/group; * *p* < 0.05; *** *p* < 0.001; ANOVA followed by Holm–Šidak multiple comparison test).

To investigate the effect of FLU on fibrotic-like processes, cell morphology (Figure 2A) and F-actin pattern (Figure 2B and Figure S4, deconvolved images) were examined. After 24 h, 20 ng/mL PDGF treatment induced cell proliferation which was prevented by FLU (Figure 2A).

In Figure 2, a wider visual field is presented to show that PDGF also caused F-actin enhancement coupled with the formation of F-actin bundles and many actin clumps (Figure 2B). More importantly, treatment with FLU resulted in prominently less fluorescence, less actin clump and stress fiber formation. Furthermore, the potent and selective S1R antagonist, NE100, suspended the effect of FLU in both measurements (Figure 2) confirming that these morphological changes are S1R-mediated. In addition to visualizing these effects, the quantification of the F-actin fluorescent signal was performed. The data are presented as integrated density, which is a commonly used metric in image evaluation. This supports the observation that FLU has a decreasing effect on F-actin (Figure 2B, right panel).

### 2.3. FLU and Other Specific S1R Agonists Mitigate the PDGF-Induced Cell Proliferation in HTM5 Cells

To further verify the effect of the S1R on cell proliferation and cytotoxicity, MTT assay was performed with other, highly specific S1R agonists: SA-4503 (Ki = 4.6 nM, IC50 = 17.4 nM) and PRE-084 (Ki = 44–53 nM, IC50 = 44 nM) [29]. None of the compounds, in any of the used concentrations, were toxic to the cells. Furthermore, each agonist inhibited PDGF-induced cell proliferation. As a control experiment, all compounds were applied to the cells in the absence of PDGF, and none of them influenced the cell number (Figure S2A), revealing that the anti-proliferative effect is linked to PDGF. Besides MTT measurement, LDH assay was used to evaluate cell death in response to the treatments. Similar to the MTT assay, cell viability was not affected either by any of the compounds (Figures S1 and S2B), so they are non-toxic to the cells.

**Figure 3:**
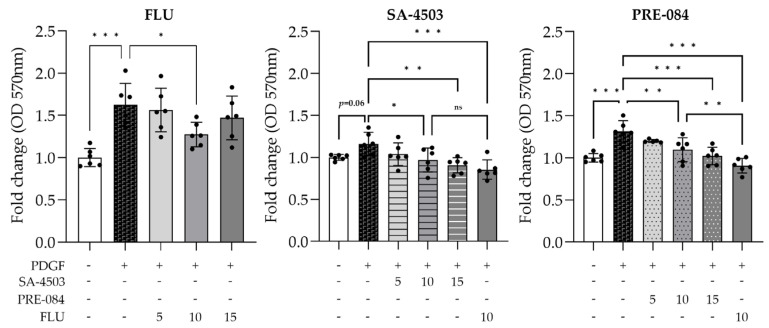
Anti-proliferative effect of various sigma-1 receptor (S1R) agonists. Fluvoxamine (FLU), SA-4503 and PRE-084 treatments ameliorate PDGF-induced HTM5 cell proliferation. (Data: mean ± SEM; n = 5–6/group; ns: non-significant; * *p* < 0.05; ** *p* < 0.01; *** *p* < 0.001; ANOVA followed by Holm–Šidak multiple comparison test).

These measurements confirmed that 10 µM FLU is most effective among S1R agonists (Figure 3), and thus it was selected for further investigations.

### 2.4. S1R Knockout (S1R^−/−^) Primary Mouse TM (pMsTM) Cells Are More Prone to Fibrotic-like Changes

#### 2.4.1. The Absence of S1R Results in More Pronounced Cytoskeletal Rearrangements in Response to PDGF, Whereas FLU Is Effective Only in Primary TM Cultures Isolated from Wild-Type Mice (WT)

**Figure 4:**
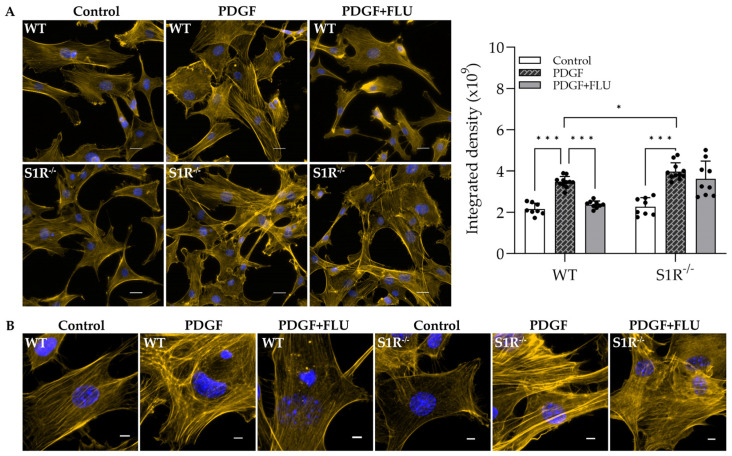
F−actin distribution in wild-type (WT) and sigma-1 receptor knockout (S1R^−/−^) primary mouse trabecular meshwork (pMsTM) cells after PDGF and fluvoxamine (FLU) treatment. (**A**) F-actin clump and filament formation are more pronounced in S1R^−/−^pMsTM cells (F-actin: yellow; nuclei: blue). FLU ameliorates cytoskeletal rearrangement in WT pMsTM but not in S1R^−/−^pMsTM cells, and PDGF induces a more robust increase in F-actin in S1R^−/−^ cells compared to WT. Quantification of fluorescence is shown as integrated density (Nikon Eclipse Ti2 microscope; magnification: 200×; scale bar: 20 µm; data: mean ± SEM; n = 8–11/group; * *p* < 0.05, *** *p* < 0.001; ANOVA followed by Holm–Šidak multiple comparison test). (**B**) Thin F-actin fibers in pMsTM cells under higher magnification prove the protective role of S1R agonist FLU in ECM rearrangement (Nikon Eclipse Ti2 microscope; magnification: 900×; scale bar: 5 µm).

Since FLU prevented F−actin enhancement, it was a further step to verify the results in primary wild-type and S1R knockout (S1R^−/−^) TM cells. In control cells (both WT and S1R^−/−^), the actin filaments are thin, with a homogenous distribution and arranged along the axial direction. Upon PDGF, F-actin enhancement and reorganization were observed with thick fibers and cross-linked network formation (Figure 4). In WT cells, FLU prevented this alteration, similarly to HTM5 cells. Interestingly, S1R^−/−^ pMsTM cells responded with a more intense and complex arrangement of thick F-actin fibers and increased clump formation compared to the WT-PDGF-induced cells (Figure 4B). As expected, FLU did not prevent PDGF-induced changes in S1R^−/−^cells.

#### 2.4.2. S1R^−/−^ Cells Respond with a Higher α-SMA Level to PDGF

**Figure 5:**
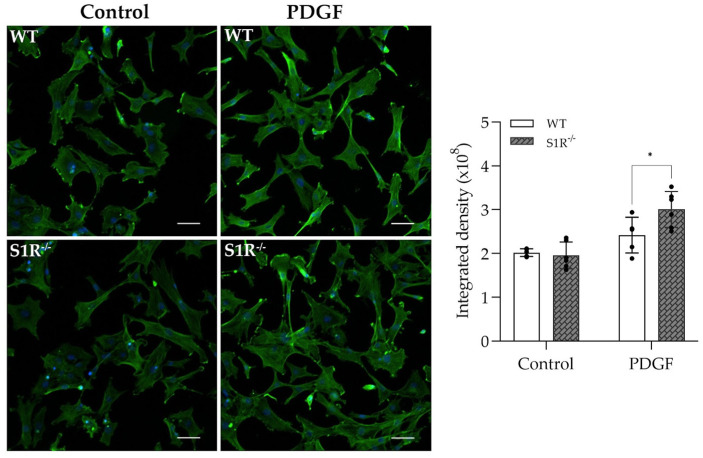
α-SMA protein level in control and PDGF-induced wild-type (WT) and sigma-1 receptor knockout (S1R^−/−^) primary mouse trabecular meshwork (pMsTM) cells. (α-SMA: green; nuclei: blue; Nikon Eclipse Ti2 microscope; magnification: 100×; scale bar: 20 µm. Result of image analysis is presented as integrated density, data: mean ± SEM; n = 5–6/group; * *p* < 0.05; ANOVA followed by Holm–Šidak multiple comparison test).

α-SMA, a key member of fibrotic processes, was also investigated in WT and S1R^−/−^ pMsTM cells. Similar to the actin cytoskeleton, S1R^−/−^ cells reacted with an enhanced α-SMA level (green fluorescence) to PDGF induction that was confirmed by the image analysis (Figure 5).

These results in pMsTM are clear evidence of the involvement of the S1R in the cytoskeletal rearrangements, and to the best of our knowledge, this study reports for the first time the anti-fibrotic property of S1R activation in the TM.

### 2.5. FLU Decreases the Production of ECM Components in HTM5

**Figure 6:**
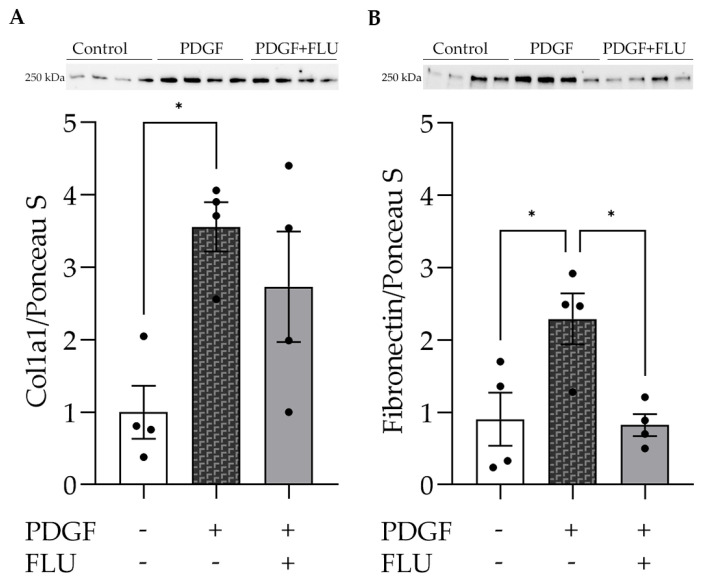
Effect of fluvoxamine (FLU) on extracellular-matrix-associated proteins. Representative images of protein level of (**A**) collagen type 1a1 (Col1a1) and (**B**) fibronectin. Protein levels were normalized to the total protein of each sample and quantified (graphs, lower panel) (Data: mean ± SEM; n = 4/group; ns: non-significant; * *p* < 0.05; ANOVA followed by Holm–Šidak multiple comparison test.)

Other key elements of the ECM, collagen 1a1 (Col1a1) and fibronectin protein levels were evaluated after profibrotic treatment with PDGF (Figure 6). Both ECM-associated proteins were highly induced, and the protective effect of FLU was also detected (the fibronectin protein level was even reduced back to the control level by FLU).

### 2.6. FLU Decreases PDGF-Induced Cell Migration of HTM5

**Figure 7:**
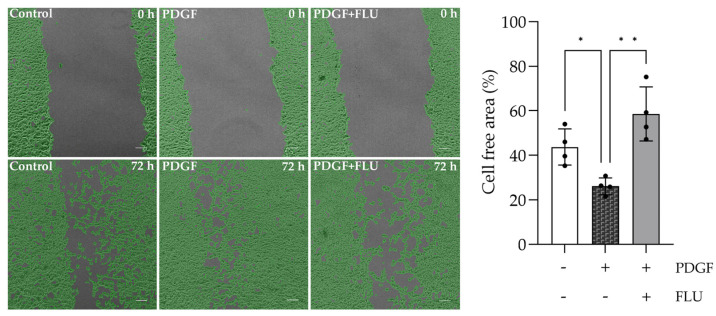
Motility analysis of human trabecular meshwork (HTM5) cells. Fluvoxamine (FLU) inhibits cell migration after 72 *h* in HTM5 cells. (Nikon Eclipse Ti2 microscope; magnification: 100×; scale bar: 100 µm; data: mean ± SEM; n = 4/group; * *p* < 0.05; ** *p* < 0.01; ANOVA followed by Holm–Šidak multiple comparison test).

To assess the effect of FLU on PDGF-induced cell migration, a scratch assay was performed in HTM5 cells (Figure 7 and Figure S5). Accelerated cell movement was observed upon PDGF, while FLU inhibited cell migration after 72 h. These results emphasize the protective effect of the S1R agonist not only in cellular structural changes but also against functional deteriorations caused by fibrosis.

### 2.7. FLU Elevates the Level of Cathepsin K and Facilitates Nitric Oxide (NO) Release in HTM5 Cells

**Figure 8:**
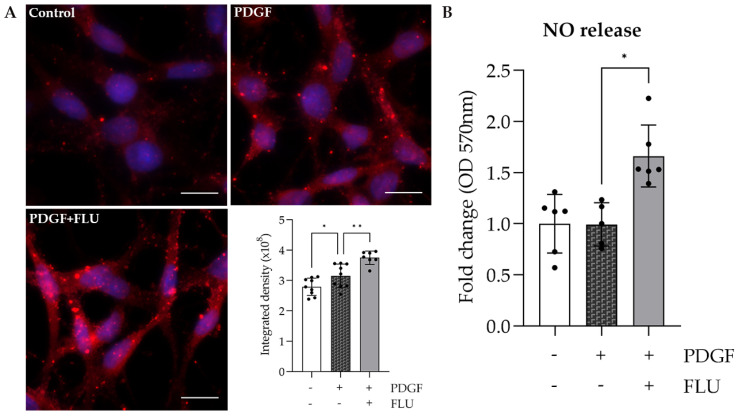
Effect of fluvoxamine (FLU) on cathepsin K and released nitric oxide (NO) levels. (**A**) FLU elevates cathepsin K level in PDGF-induced HTM5 cells (cathepsin K: magenta; nuclei: blue; Nikon Eclipse Ti2 microscope; magnification: 600×; scale bar: 20 µm; data: mean ± SEM; n = 7–9/group; * *p* < 0.05; ** *p* < 0.01; ANOVA followed by Holm–Šidak multiple comparison test). (**B**) FLU increases NO release in PDGF-activated HTM5 cells (data: mean ± SEM; n = 5–6/group; * *p* < 0.05; ANOVA followed by Holm–Šidak multiple comparison test).

To test whether FLU can also facilitate ECM degradation, cathepsin K (CTSK) was measured. CTSK is a lysosomal cysteine protease and a potent collagenase that has been strongly linked to IOP regulation [30,31]. Immunofluorescent images revealed that FLU further increases the PDGF-elevated CTSK level (Figure 8A), which is a novel effect of FLU that could partly explain its anti-fibrotic (ECM-degrading) effect.

NO has also been reported to affect TM contractility and thus facilitate outflow [32,33,34]. Fluorimetric assay with an NO-binding fluorescent dye revealed that FLU increases NO production in HTM5 cells (Figure 8B), which is also a new effect of the S1R agonist.

## 3. Discussion

The protective role of the S1R in the central nervous system is proven by a wide range of studies [35,36,37,38,39]. Moreover, its functional importance in the retina has been emphasized as well [16,17,18,19,20,21,23,40]. Surprisingly enough, there is a lack of information regarding its function in the TM. There is only one study reporting that S1R activation protects against high-pressure-induced damage in human TM cells [41]. In addition, to confirm the presence of the S1R in HTM5 cells, we proved that the S1R resides mainly in the ER membrane, but it is also found in the cytoplasm of TM cells (Figure 1).

The main aim of this study was to investigate the effect of FLU on fibrotic-like changes in the TM, including cytoskeletal rearrangement, proliferation, migration and profibrotic proteins. Prior to these investigations, we demonstrated that FLU enters the living cells and does not change the protein level of the receptor (Figure 1). We also proved that FLU is a safe and non-toxic compound to treat TM cells. Based on all viability assays and our pilot experiments, we decided to use 10 µM of FLU throughout our study.

PDGF is a well-known inducer of tissue fibrosis, promoting cell proliferation and migration, as well as other ECM-related events [13,42,43]. It is known that the actin cytoskeleton is a popular therapeutic target, since structural changes in TM tissue result in increased stiffness and lead to increased IOP. Thus, we tested the effect of FLU on F-actin architecture, together with the NE100 antagonist. The massive F−actin rearrangement and morphological changes induced by PDGF were prevented by FLU and reversed by the antagonist NE100. All these results revealed that the S1R has an important role in the ECM remodeling of HTM5 cells.

More importantly, the knockout studies clearly confirmed the protective role of the S1R in PDGF-induced cytoskeletal rearrangement. The formation of actin clumps and the thickness of actin fibers were markedly more pronounced in the absence of the receptor. Of note, FLU could ameliorate this phenomenon only in WT primary cells, thus suggesting a direct beneficial effect of S1R activation. Furthermore, besides F-actin, a PDGF-induced increase in the contractile activity-regulating protein α-SMA [44,45] was also more prominent in the S1R^−/−^ cells. All of this suggests a protective role of the S1R in the TM against contraction-altering processes as well; however, specific studies have to be performed to justify this.

One may speculate that FLU may also decrease ECM production and deposition by interacting with other ECM elements, such as Col1a1 and fibronectin. Western blot results revealed that the increase in these important fibrosis elements was prevented by FLU, which can represent another side of the complex protective effect that we propose.

Further explanation for the FLU effect on the cytoskeleton is facilitating ECM degradation. In addition to matrix metalloproteases, a well-known group of enzymes responsible for ECM degradation, cathepsins, have been recently reported to participate in ECM remodeling and decomposition [31,46]. Elevated CTSK was shown to decrease actin stress fibers and the ECM element level [30,31]. We found here that FLU remarkably elevated the CTSK level in HTM5 cells (Figure 8A), which can in turn result in decreased ECM deposition. We propose that an F-actin (Figure 2B) and fibronectin decrease (Figure 6) may also be the result of this ECM degrading effect of FLU. Of note, to confirm the reduction in Col1a1, further investigations are needed.

Importantly, ECM plays a critical role in IOP homeostasis [47]. We hypothesize that decreased ECM remodeling and deposition by FLU may reduce TM outflow drainage resistance resulting in IOP reduction. In a study carried out in porcine eyes, it was shown that the inhibition of CTSK significantly increased IOP [31]; thus, it is very likely that FLU-mediated CTSK activation can lead to an IOP decrease. Taken together, FLU could be a potential candidate to reduce IOP in ocular hypertension.

Another important mediator of IOP through facilitating aqueous humor outflow is NO [32,48,49,50]. Its protective effect has been proven on the posterior eye structures related to glaucoma pathogenesis, including the optic nerve and ocular blood vessels [50,51,52,53,54]. Interestingly, we showed in HTM5 cells that FLU increases NO release. This phenomenon could also be contributing to IOP lowering and thus making FLU a more valuable agent.

The inhibitory effect of FLU on actin polymerization, cell migration and invasion has been demonstrated in the brain on human glioma cells [55], and therefore its protective effect in other tissues in this manner was expected. We showed with various models that FLU has anti-fibrotic effects in HTM5 cells, and in addition, it can enhance NO production, thus further increasing the tissue protection assured by FLU. We also showed that the loss of the S1R exhibits more pronounced fibrotic-like alterations indicating an important and protective role of the S1R in the regulation of TM stiffness and thus IOP homeostasis.

Tissue fibrosis is a highly complex process. Identifying all elements targeted by FLU, or describing each element of the protective mechanism through S1R agonism, is beyond the scope of this study. However, these results serve as predictors for our future plans, which include an in vivo investigation using a mouse glaucoma model. The aim of this investigation is to ameliorate TM fibrosis and subsequently lower intraocular pressure.

In summary, our findings provide evidence of the presence of the S1R in both the endoplasmic reticulum and the cytoplasm of human TM cells. As pioneers in this research, we demonstrate that S1R agonists have a protective effect on TM cells, counteracting fibrotic changes induced by PDGF. These changes include inhibiting cell proliferation, the migration and accumulation of ECM elements and preventing morphological and actin cytoskeletal alterations. More importantly, we confirm the protective role of the S1R in fibrosis by conducting experiments on primary S1R knockout TM cells (S1R^−/−^). Additionally, we present a novel finding that FLU promotes the release of NO, which has been reported to exhibit protective effects on the eye and increase AH outflow facility [32,48,49,50,51,52,53,54].

## 4. Materials and Methods

### 4.1. Materials

All reagents and chemicals were purchased from Merck KGaA (Darmstadt, Germany) unless stated otherwise, and all standard plastic laboratory equipment was purchased from Sarstedt (Nümbrecht, Germany).

### 4.2. Experimental Animals and Primary Mouse Trabecular Meshwork (pMsTM) Cell Isolation

Three-month-old male C57BL/6J mice were purchased from Animalab (Budapest, Hungary). One/The first pair of S1R knockout (S1R^−/−^) mice were kind gift of Adrian Y.C. Wong, University of Ottawa, and were then bred and housed at our institute. Mice were housed in groups of three under a 12 h light–dark cycle at 22 ± 2 °C with free access to standard rodent diet and tap water. pMsTM cells were isolated on a magnetic bead−based method as described previously [56]; briefly, 2.0–2.5 µm in diameter magnetic polystyrene beads (PM-2.5, Kisker Biotech GmbH, Steinfurt, Germany) were injected intracamerally, and mice were sacrificed after 7 days. Eyes were carefully dissected under microscope, and anterior segments pooled from 3–4 mice were enzymatically digested with 4 mg/mL collagenase II (10566-016 Gibco, Thermo Fisher Scientific, Waltham, MA, USA) for 3 h at 37 °C, 5% CO_2_. After repeated washings with PBS containing 0.5% bovine serum albumin (BSA, A2153 Sigma-Aldrich, St. Louis, MO, USA) and 2 mM EDTA, cells were loaded on MACS column (130-042-801 Miltenyi Biotech, Bergisch Gladbach, Germany); then, they were eluted after removing the column from the magnetic fields and washed again. pMsTM were cultured in regular Dulbecco’s Modified Eagle’s Medium (DMEM, 10566-016 Gibco) supplemented with 10% fetal bovine serum (FBS, 10500064 Gibco) and 1% penicillin/streptomycin (15140-122 Gibco). Cells were incubated at 37 °C in a humidified atmosphere of 5% CO_2_ and 95% air. Before experiments, cells were plated in media containing 1% FBS (10500064 Gibco) for 24 h and then treated with either 20 ng/mL PDGF ( 520-BB, R&D Systems, Minneapolis, MN, USA) alone or in combination with 10 µM FLU (F2802, Sigma-Aldrich) in fresh media supplemented with 1% FBS (10500064 Gibco) to avoid cell detachment. After the treatment period, cells were washed, and F-actin and α-SMA were detected immunocytochemically.

### 4.3. Human Cell Culture

Human transformed TM cells from a non-glaucomatous donor (HTM5) were developed and obtained from Abbot Clark (University of North Texas—Health Science Center, TX, USA) [57] and kindly provided by Xavier Gasull (University of Barcelona) for this study. HTM5 cells were cultured in DMEM supplemented with 10% FBS, 1% penicillin/streptomycin and 1% L-glutamine (all from Gibco, as mentioned above). Cells were incubated at 37 °C in a humidified atmosphere of 5% CO_2_ and 95% air. For cell characterization, HTM5 were treated with 100 nM dexamethasone (1177-87-3, Sigma-Aldrich, St. Luis, MO, USA) for one week and then immunolabeled for myocilin (ab41552, Abcam, Cambridge, UK, 1:50), α-SMA (124964 Abcam, 1:200) and fibronectin (ab2413, Abcam, 1:100) at 4 °C, overnight, using standard protocol detailed below.

### 4.4. Immunocytochemistry

HTM5 cells were plated at a density of 150,000 cells/well on 0.1% gelatin (G1393 Sigma-Aldrich) coated tissue culture chambers and serum-starved for 24 h before each experiment. For S1R localization, cells were washed with PBS, fixed with ice-cold methanol, washed, blocked with 5% BSA for 1 h at room temperature (RT) and then incubated with the rabbit anti-S1R antibody (53852 Abcam, 1:50, at 4 °C, overnight) followed by the second anti-rabbit Alexa568 secondary antibody (A11036 Life Technologies, 1:500, 1 h, RT). After repeated washings, cells were labeled for ER marker Grp94 (MA3-016 Invitrogen, 1:250, 2 h, RT) followed by Alexa488 anti-mouse antibody (A11001 Life Technologies, 1:500, 1 h, RT).

For visualization of fibrosis-related proteins, cells were treated for 24 h with platelet-derived growth factor (PDGF; 20 ng/mL, 520-BB R&D Systems, Minneapolis, MN, USA) alone or in combination with Fluvoxamine maleate (FLU; 10 µM, F2802 Sigma-Aldrich, St. Luis, MO, USA), then washed and fixed in 4% paraformaldehyde (30525-89-4 Sigma-Aldrich, 10 min, RT), washed again, permeabilized with Triton X-100 (9036-19-5 Sigma-Aldrich, St. Louis, MO, USA, 15 min, RT) and blocked with 5% BSA for 1 h at RT. Repeated washes with PBS were followed by incubation with mouse anti-α-SMA (124964 Abcam, 1:200) or CTSK7 (188604 Abcam, 1:100), both overnight at 4 °C, washings and incubation with Alexa488 anti-mouse (A11001 Life technologies) and Alexa568 anti-rabbit (A11036 Life technologies) antibodies, respectively, both used in a dilution of 1: 500, 1 h, RT.

Cytoskeleton rearrangements were visualized by F-actin staining using Alexa546-phalloidin (1:40; A22283 Invitrogen) applying the same washing, fixation and blocking steps, after treatments for 24 h with PDGF (20 ng/mL) alone or in combination with FLU (10 µM) with or without NE100 (3 µM, 3133 Tocris Bioscience, Bristol, UK).

At the end of each protocol, slides were washed with PBS, and nuclei were stained with Hoechst (33342 Invitrogen, 5 µM, 10 min, RT). Finally, after rinsing with PBS, coverslips were mounted onto slides using ProLong Anti-Fade (P36980 Life Technologies, Waltham, MA, USA).

Cy5-FLU was manufactured by Vichem Ltd. (Budapest, Hungary) for these experiments, and it was used for live cell imaging. Cells were plated on gelatin-coated culture slides as described above. A total of 500 nM of Cy5-FLU was added to the cell culture, the chamber was placed in a microscope top-up incubator, and images were taken after 4 h.

All images were acquired using Nikon Ti2 inverted microscope (Nikon Corporation, Tokyo, Japan) equipped with a 10×, 20×, 40× and 60× oil immersion objective (Plan Apo lambda, N.A. 1.4) plus a 1.5× intermediate magnification and a cooled sCMOS camera (Zyla 4.2, Andor Technology). Image analysis was performed using Nikon software (AR 5.21.03 software version). For proper and accurate quantification of fluorescence, integrated density values were calculated by multiplying the mean fluorescence intensity of the region of interest (ROI) by the area of the ROI. Representative images were prepared from original images after background subtraction using background ROI and cropping 700 × 700 pixels images. Colocalization analysis was performed on deconvolved image stacks using Huygens Essential 21.10 (Scientific Volume Imaging, Hilversum, The Netherlands) [58], and Pearson correlation coefficient, describing the correlation of the intensity distribution between the channels, and Spearman’s coefficient, which is equal to Pearson’s but is based on the intensity ranks instead of intensity values, were determined.

### 4.5. Cell Proliferation Assay

HTM5 cells were plated in serum-free fresh medium in 96-well plates (Sarstedt) at a density of 40 000 cells/well (n = 6 wells/group) and treated with 20 ng/mL of PDGF, 5–15 µM FLU (PDGF + FLU), 5–15 µM SA-4503 (PDGF + SA; SA−4503: 165377-44-6 Tocris Bioscience, Bristol, UK) and 5–15 µM PRE-084 (PDGF + PRE; PRE−084: P2607 Sigma-Aldrich, St. Louis, MO, USA) for 24 h. Control cells were treated with vehicle (HCl) alone. After the treatment period, cells were incubated with methyl-thiazoletetrazolium (MTT, M6494 Invitrogen, Carlsbad, CA, USA) for 4 h followed by solubilization in DMSO-ethanol (1:1). The formation of water-insoluble formazan was determined by measuring optical density at 570 nm in a Plate CHAMELEON™ V Fluorometer-Luminometer-Photom reader (Hidex, Turku, Finland). Lactate dehydrogenase (LDH) assay (C20300, Invitrogen, Carlsbad, CA, USA) was performed from the supernatant of the cells according to the manufacturer’s protocol, and samples were measured in the same way as in case of MTT.

### 4.6. Cell Migration Assay

The mechanical scratch wounding of confluent monolayers served as a model to study cell migration. HTM5 cells were grown until confluence, and the cell monolayer was scraped in a straight line with a p200 pipet tip to create a “scratch”. Cells were washed with PBS to remove debris. Then, wells received serum-free medium containing PDGF (20 ng/mL) or FLU (10 µM) treatments, while controls received vehicle alone. Images were taken from the scratched area every hour for 24 h using Nikon Eclipse Ti2 microscope (Nikon Corporation, Tokyo, Japan). The percentage scratch area at each time was calculated using Nikon NIS-Elements Analysis Software (v.5.19, Nikon Corporation, Tokyo, Japan). The cell-free area was calculated as % of 0 h in case of each well.

### 4.7. NO Measurements

To measure the released NO, a reliable method, DAF-FM diacetate (D23844 Invitrogen), was used. All steps followed the manufacturer’s instructions; briefly, cells were plated in a black 96-well plate, and after the regular starvation period, cells were treated with PDGF and PDGF + FLU. Then, cells were washed and incubated with 5 µM DAF-FM, and fluorescence was measured using microplate reader (ClarioStar, BMG Labtech, Ortenberg, Germany).

### 4.8. Subcellular Fractionation

HTM5 cells were plated in 10% FBS containing complete fresh medium complemented with 1% Penicillin/Streptomycin (15140-122; Gibco) in T25 flasks (Sartstedt) at density of 1,000,000 cells/flasks (n = 4) and were serum-starved for 24 h after attaching. For S1R subcellular localization, cells were washed with PBS and harvested with 0.25% Trypsin-EDTA (25200-072; Gibco). The subcellular fractionation was performed using Subcellular Protein fractionation kit (78840; Thermo Fischer) following its recommended protocol. The subcellular localization of S1R was detected from 10 µg protein samples by Western blot.

### 4.9. Western Blotting

All reagents for Western blot analysis were obtained from Bio-Rad Laboratories (Hercules, CA, USA). After treatments, cells were rinsed with ice-cold PBS and were lysed in a lysis buffer of 1.7 mg/mL aprotinin, 1 M tris base, 5 mg/mL leupeptin, 0.5 M EGTA, 0.25 M NaF, 1% Triton X, 0.5 M PMSF and 0.5 M Na_3_VO_4_ with sonication and vortex alternatively. Lysates were then centrifuged at 13,000 rpm for 10 min at 4 °C and transferred to a clean tube, and protein concentration was determined with Protein DC kit (5000111 Bio-Rad Laboratories, Inc.). Samples were mixed with 4× loading buffer (161-0747, Bio-Rad) and then heated at 95 °C for 5 min. Total protein (10 µg) was loaded to polyacrylamide gradient gels (4–20%) and transferred to nitrocellulose membranes. Membranes were blocked by 5% non-fat milk or 5% BSA in TBS at RT for 1 h and then incubated with primary antibodies (Myocilin, ab41552 Abcam, 1:500; S1R, sc137075 Santa Cruz, 1:1000; Fibronectin, ab2413 Abcam, 1:5000; Col1a1, 72026 Cell Signaling, 1:1000) at 4 °C overnight. The membranes were washed and then incubated with the corresponding horseradish peroxidase-conjugated secondary antibodies (1:3000, Cell Signaling). The signals were detected using Luminata Forte (Millipore, Billerica, MA) by ChemiDocTM MP Imaging System (Bio-Rad) and analyzed using Image Lab software (version 6.1.0, Bio-Rad Laboratories). After background subtraction, integrated optical densities of bands of interest were factored for Ponceau S staining to correct for variations in total protein loading. Each blot was normalized to an internal control so that bands on separate blots could be compared.

### 4.10. Statistical Analysis

Statistical analyses were performed using GraphPad Prism software version 6.01 (GraphPad Software Inc., San Diego, CA, USA). To test if the values were from a Gaussian distribution, a Kolmogorov–Smirnov normality test was performed. Data were analyzed by one-way ANOVA followed by Holm–Šidak multiple comparison post hoc test for all parametrical comparisons or, in the case of non-parametric data, by Kruskal–Wallis ANOVA of ranks. Significance was set a priori at *p* < 0.05, corrected for multiple comparisons.

## 5. Conclusions

In this study, we confirmed the presence of the S1R in TM cells and determined its subcellular localization. We provided evidence supporting the protective effect of the S1R agonist FLU on various ECM-related functions in response to profibrotic stimuli. Furthermore, we demonstrated that the S1R plays a protective role in TM fibrosis, which can be attributed to its ability to degrade the ECM and facilitate the release of nitric oxide (NO).

In conclusion, we strongly believe that directly targeting the S1R in TM fibrosis offers an attractive strategy for therapeutic intervention in POAG or other TM-associated fibrosis-related eye disorders.

## 6. Patents

Patents: “Novel use of sigma-1 receptor agonist compounds”, US20190209575A1 [15]. The invention is directed to compositions and methods for the prevention and/or treatment of progressive fibrosis in various organs.

## Data Availability

Not applicable.

## Supplementary Materials

The following supporting information can be downloaded at https://www.mdpi.com/article/10.3390/ijms241411646/s1.
