# Supplementary material for: Sigma-1 Receptor Agonist Fluvoxamine Ameliorates Fibrotic Response of Trabecular Meshwork Cells
Source: Int J Mol Sci. 2023 Jul 19;24(14):11646. doi: 10.3390/ijms241411646 (PMC10380218; doi:10.3390/ijms241411646)
Supplement: Supplementary file 1 [file ijms-24-11646-s001.zip › ijms-2456396-supplementary.pdf]

International Journal of *Molecular Sciences*

MDPI

Supplementary materials

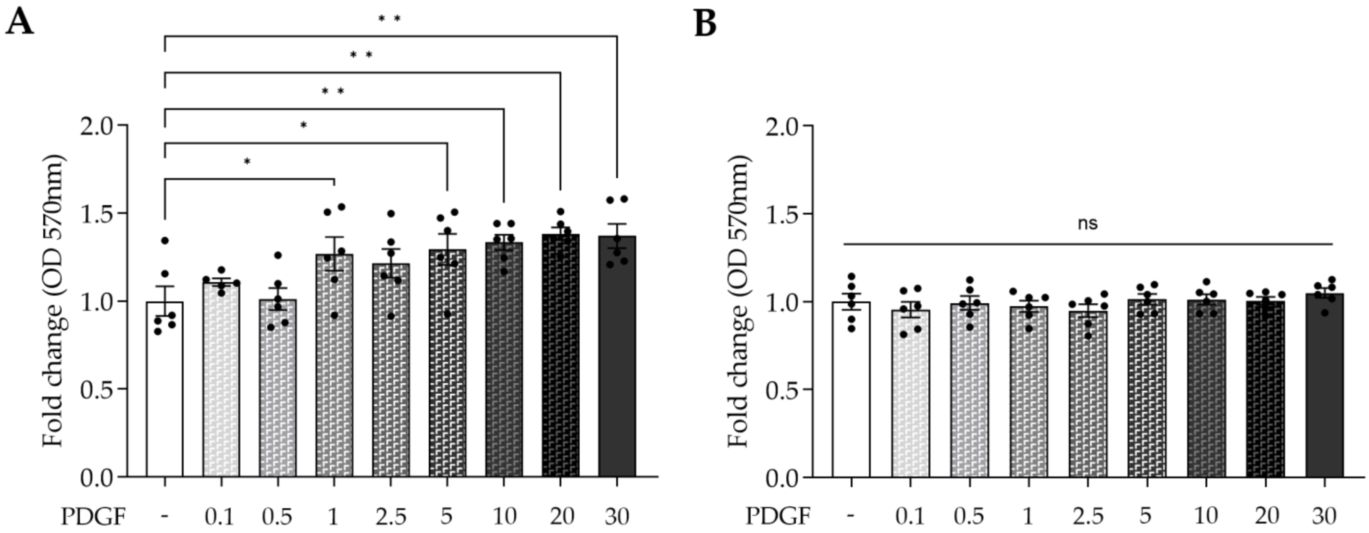

Figure S1. Dose−response curve of PDGF on human trabecular meshwork (HTM5) cells. (A) Cell proliferation MTT and **(B)** cytotoxicity LDH assay of PDGF−induced HTM5 cells. PDGF has proliferative but non−toxic effect on HTM5 cells. (Data: mean ± SEM; n=5−6/group; ns: non−significant; *p<0.05; **p<0.01; ANOVA followed by Holm−Šidak multiple comparison test).

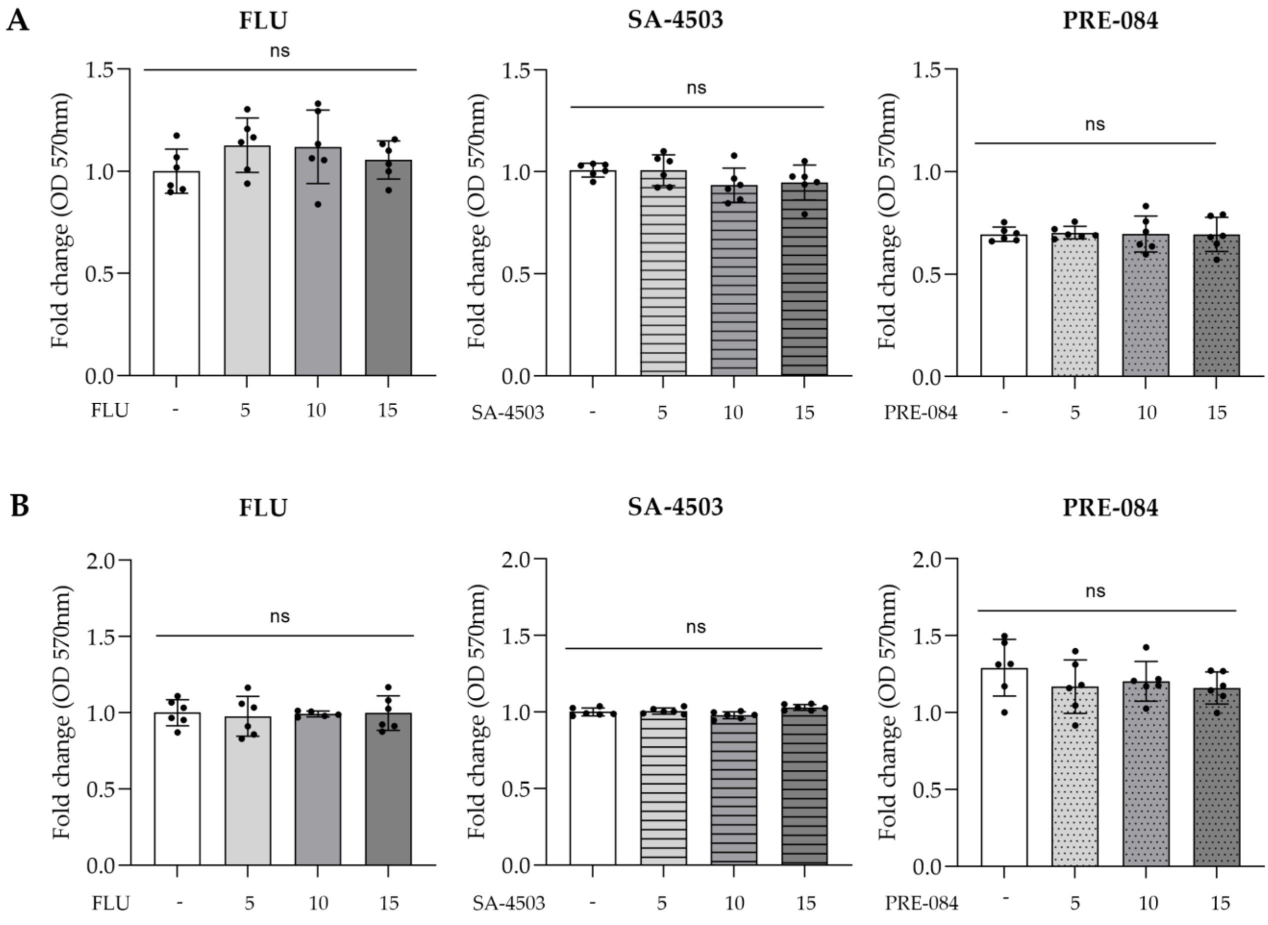

Figure S2. Dose−response of various Sigma−1 receptor (S1R) agonists. (**A**) Cell proliferation MTT and (**B**) LDH cytotoxicity assay shows none of the S1R agonists has proliferative or cytotoxic effect in itself on HTM5 cells. (Data: mean ± SEM; n=5−6/group; ns: non−significant; ANOVA followed by Holm−Šidak multiple comparison test).

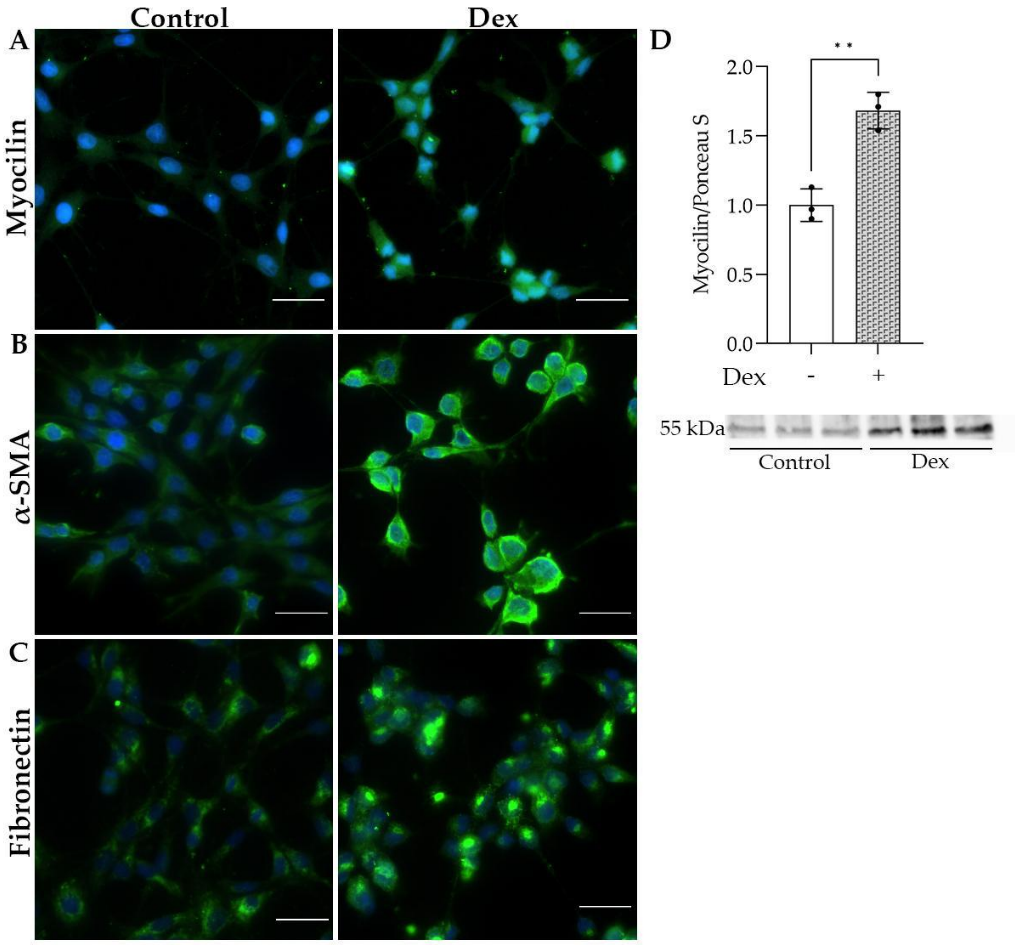

Figure S3. **Dexamethasone (Dex) induces (A) myocilin and (B) α−SMA and (C) fibronectin in HTM5 cells**. **(D)** Dex induced myocilin expression is detectable with Western blot (Nikon Eclipse Ti2 microscope; magnification: 400x; scale bar: 20 μm; data: mean ± SEM; n=3/group; **p<0.01; unpaired *t−test*).

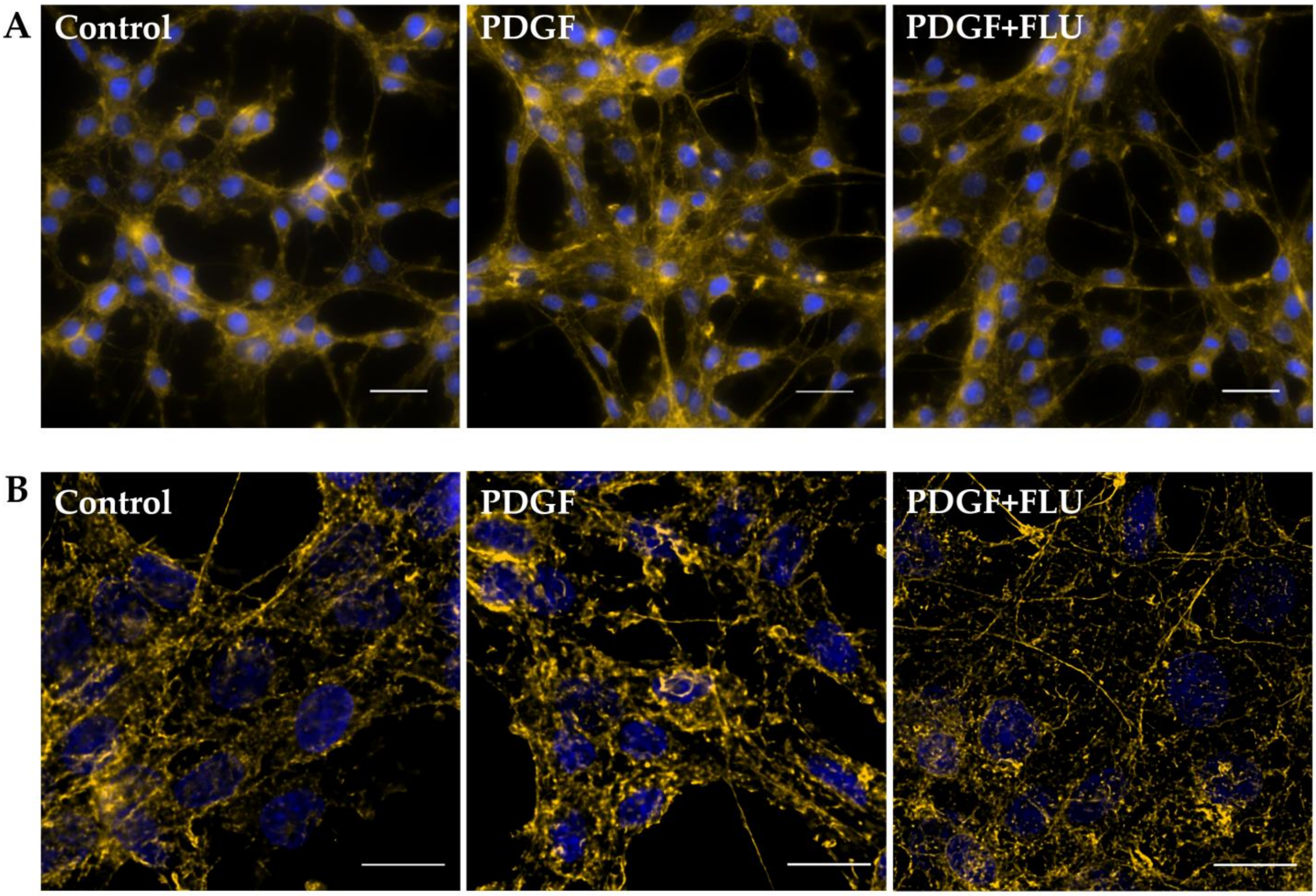

Figure S4. Higher magnification of F−actin formation in human trabecular meshwork (HTM5) cells during PDGF activation and fluvoxamine (FLU) treatment. (**A**) PDGF−induced F−actin filament and clump formation is inhibited by FLU treatment. (**B**) Deconvolved cropped images show prominent F-actin filaments and actin clump accumulation after PDGF treatment. These massive changes in fiber structures were suppressed by FLU treatment. (Nikon Eclipse Ti2 microscope; F-actin: yellow; nuclei: blue; magnification: 600x; scale bar: (A) 20 µm; (B) 10 µm).

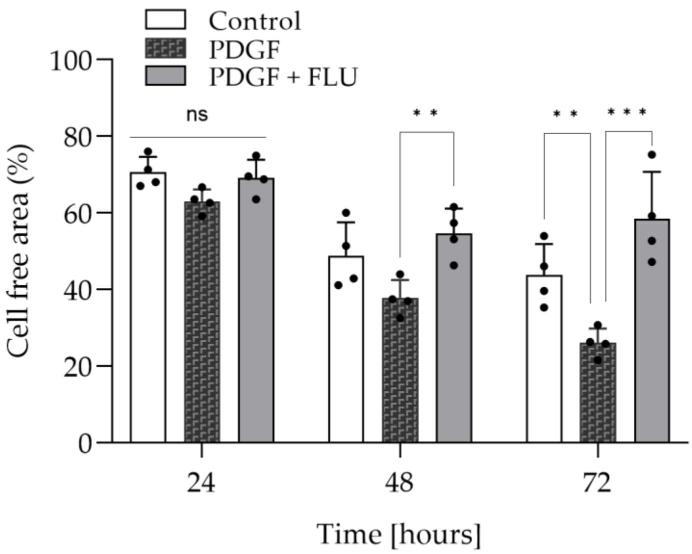

Figure S5. Cell migration of human trabecular meshwork (HTM5) cells. The analysis shows FLU significantly inhibits cell migration after 48 and 72 hours in PDGF−induced cells. (Data: mean ± SEM; n=4/group; *p<0.05; **p<0.01; ***p<0.001; ANOVA followed by Holm−Šidak multiple comparison test).
